# Verifications in the October Number

**Published:** 1890-11

**Authors:** Wm. Jefferson Guernsey


### VERIFICATIONS IN THE OCTOBER NUMBER.

Editors Homœopathic Physician:

When I sent you the little article which appeared on page 475 of the October number, I did not deem it worthy of a *name*, but observe that you have nominated it "verifications." Certainly if the child had to have a name this was an appropriate one, but now comes a subdivision, and the writer is startled on discovering that (according to the first caption that met his gaze) he had written on "Hydrophobia" instead of *Tetanus*, as he had supposed. The contagiousness of the malady is obvious, from the fact that he felt himself becoming "mad" at once.

You doubtless thought that by thus giving it a name of a disease which was *similar* you would render the article more homœopathic. Be that as it may, its action upon myself was quite remarkable if not soothing. However, I must destroy your poetic idea by asking that you call attention to the fact that I am not responsible for an error which places me in a rather ridiculous light. I am, yours respectfully,

Wm. Jefferson Guernsey.

[We regret exceedingly the error of which Dr. Guernsey complains. Our deliberate intention was to put the word *Tetanus* at the head of the paragraph, but our thoughts becoming drawn to the question of hydrophobia by the doctor's suggestive case we mechanically wrote that word.—Eds.]

